# Notes and News

**Published:** 1900-10-20

**Authors:** 


### Notes and News.

We regret to announce the death of Lieut.-Col. S. W. Wilkinson, who was for 52 years Hon. Secretary to the Stockport Infirmary.

The study of tropical diseases seems to have gained popularity on the Continent as well as in England. The Bordeaux University has made provision for lectures on the subject, and so also has the University of Utrecht.

The secretary of the Hospital Saturday Fund states that in reply to inquiries made by the fund he has been informed that during the past year 46 institutions show a decrease in funds to the extent of £24,582. Less than half the institutions appear to have answered the queries addressed to them.

The care of lunatics forms an important part of the future programme of the London County Council. They are about to spend £1,000 in formulating a practical scheme for providing another asylum, and securing plans for the same. They also propose establishing an epileptic colony for male patients. The boarding out of lunatics and the establishment of receiving houses for the insane, to take the place of lunatic wards in workhouse infirmaries, is also under consideration.

A most unfortunate affair occurred recently at Leicester, resulting in serious injury to Dr. Clarke, of that town. It appears that for some time past a man, who many years ago was confined in a lunatic asylum, has used threats towards Dr. Clarke and his son, also a medical man, for some alleged grievance, and last Monday this man, who had been watching for Dr. Clarke, on seeing him leave his carriage went up close behind and fired at him with a revolver. The shot took effect near the spine.

The foundation-stone of a sanatorium to be erected at Delamere, Cheshire, in connection with the Liverpool Hospital for Consumption, was laid last Saturday by the Earl of Derby. The sanatorium, which is intended to accommodate fifty patients, and will cost upwards of £15,000, is being presented to the city of Liverpool by Lady Wilcox and Mr. W. P. Hartley, of Aintree. The building will contain no rooms for patients except the bedrooms and the dining-hall, it being intended that the patients shall live in the open air except when they are sleeping or taking their meals.

Dr. Arthur Shadwell has been engaged by the Metropolitan Asylums Board to advise them in matters concerning plague.

General Sir F. Grenfell has sent his second contribution towards the maintenance of the cancer research department at the Middlesex Hospital.

The annual meeting of the Metropolitan Hospital Saturday Fund will be held on Saturday, October 27th, at 3 p.m., when the result of this year's collection will be announced.

Messrs. Smith, Elder & Co. announce a second edition of the late E. J. Chance's "Bodily Deformities," volume I., edited by John Poland, F.R.C.S. The hitherto unpublished volume II. will also be edited by Mr. Poland.

Sydney may now be regarded as quite free of the plague. Now that alarm is practically over, some of the inhabitants are beginning to declare that unnecessary precautions were taken in some cases by the Government, and one or two business firms are contemplating an action to recover damages due to interference.

The beautifully situated city of Srinagar, in Cashmere, has lately been visited by a good deal of cholera. The native portion of the city is very filthy, and as most of the dwellings of the English residents take the form of houseboats, they stand in special danger from water-borne diseases. The last two visitations of cholera in 1888 and 1892 carried off about 10,000 victims each time.

Some alarm was occasioned a few days ago by a report that a case of plague had appeared at Limehouse. The Local Government Board have, however, been able to deny the truth of the report officially, Dr. Klein having made a bacteriological examination, which shows that the patient is not suffering from bubonic plague. Every precaution is being taken to detect any case of plague which may appear in London.

Mr. Arthur Pierce, Secretary of the Royal Sea-Bathing Infirmary, has resigned his post after an occupation of eighteen years. The directors have awarded him £400 on his retirement. Mr. Pierce is now Secretary Superintendent at the Hereford General Infirmary. Mr. Nash, who held a post in the treasurer's office at St. Thomas's Hospital, has been selected as secretary in place of Mr. Pierce at Margate.

An arrangement has been very properly made by the Metropolitan Asylums Board to receive patients suffering from small-pox from certain districts adjacent to but outside the metropolitan area, such as Gravesend, Orsett, and West Ham, and also from Her Majesty's ships at Sheerness and Chatham. From all these places it is arranged that patients suffering from small-pox may be received, at so much a case, on the understanding that a suspension of the arrangement may become necessary should any extensive outbreak of the disease occur in London. Clearly, the Metropolitan Asylums Board cannot relieve these outside bodies of their responsibility, nor can they provide for outsiders to the exclusion of Londoners; but it is much to the advantage of the community that in times when only a few cases occur these should be dealt with at one place, and that the district should not be dotted over with a number of separate small-pox hospitals, each serving as a centre of infection while doing but little useful work.

